# Association of regional white matter hyperintensity volumes with cognitive dysfunction and vascular risk factors in patients with amnestic mild cognitive impairment

**DOI:** 10.1111/ggi.14211

**Published:** 2021-06-09

**Authors:** Kentaro Hirao, Fumio Yamashita, Shu Sakurai, Akito Tsugawa, Rieko Haime, Raita Fukasawa, Tomohiko Sato, Hidekazu Kanetaka, Takahiko Umahara, Hirofumi Sakurai, Haruo Hanyu, Soichiro Shimizu

**Affiliations:** ^1^ Department of Geriatric Medicine Tokyo Medical University Tokyo Japan; ^2^ Department of Ultrahigh Field MRI, Institute for Biomedical Sciences Iwate Medical University Iwate Japan

**Keywords:** cystatin C, deep white matter hyperintensities, mild cognitive impairment, periventricular hyperintensities, regional white matter hyperintensities

## Abstract

**Aim:**

White matter hyperintensities (WMH) obtained by magnetic resonance imaging (MRI) have been reported to promote neurodegeneration and cognitive decline in patients with mild cognitive impairment (MCI). However, little is known about the association between regional WMH (rWMH) and cognitive dysfunction in MCI. We hence investigated the associations between rWMH volumes and cognitive dysfunction in MCI.

**Methods:**

Thirty‐eight subjects with amnestic MCI were analysed. The volumes of periventricular hyperintensities (PVH) and deep WMH (DWMH) were measured on a T2‐FLAIR MRI using a 3D‐slicer, and regional PVH and DWMH (rPVH and rDWMH) volumes were calculated. The associations of rPVH and rDWMH volumes with cognition and blood levels of various molecules were investigated. Furthermore, rPVH and rDWMH volumes were compared between MCI with vascular risk factors, such as hypertension, diabetes mellitus (DM), and dyslipidemia, and those without these risk factors.

**Results:**

rPVH volume (bilateral cornu frontale, pars parietalis, and cornu occipitale) positively correlated with Trail Making Test‐A/B scores and CysC level, whereas rDWMH volume did not correlate with any of the items. rPVH volumes (right cornu frontale, bilateral pars parietalis and cornu occipitale, and right pars temporalis) and rDWMH volumes (left frontal and parietal lobes) were significantly larger in MCI patients with DM than in those without.

**Conclusions:**

PVH volumes (bilateral areas of cornu frontale, pars parietalis, and cornu occipitale) were closely associated with attention and executive dysfunction. Serum CysC level and DM were associated with WMH volume, suggesting that CysC level and DM might be important markers for determining treatment strategies for white matter abnormalities in MCI. **Geriatr Gerontol Int 2021; 21: 644–650**.

## Introduction

The contribution of white matter abnormalities to clinical symptoms, such as cognitive dysfunction and geriatric depression, and the pathogenesis of Alzheimer's disease (AD) has been increasingly recognized.^1^, ^2^, ^3^ White matter hyperintensities (WMH) displayed by magnetic resonance imaging (MRI) have been shown to be a risk factor for the conversion from mild cognitive impairment (MCI) to AD. Several recent studies have reported associations between WMH, such as of the periventricular and parietal white matter regions, and the risk of the future development of AD, although MCI is a heterogeneous disorder with a variety of clinical outcomes (amnestic and nonamnestic MCI, etc.).^4^, ^5^ However, most of these studies have assessed WMH by visual rating, which is not very precise. We recently reported the association of periventricular hyperintensity (PVH) volume with attention and executive dysfunction in amnestic MCI patients.^6^ However, we did not assess the associations between WMH in specific locations and cognitive dysfunction. In the present study, we hence calculated regional WMH (rWMH) volumes using a parcellated template, and investigated the association between rWMH volumes and cognitive dysfunction, blood levels of various molecules, and the presence of vascular risk factors, such as hypertension (HT), diabetes mellitus (DM), and dyslipidemia (DL), in amnestic MCI patients.

## Materials and methods

### Subjects

Outpatients (aged > 60 years and <90 years) who were enrolled at the memory clinic or outpatient clinic of Tokyo Medical University were prospectively recruited between 2015 and 2018. Written informed consent was obtained from all subjects before the study. The study design was approved by the ethics review board of Tokyo Medical University. Data from 38 subjects with amnestic MCI and from 10 normal control (NC) subjects, who were spouses of the MCI subjects or who were being followed at the outpatient clinic but had no memory complaints, were medically stable, and had Mini Mental State Examination (MMSE) scores of 28 and above, were analysed. All patients underwent detailed general physical, neurological, and psychiatric examinations, and extensive laboratory tests, including MRI and single‐photon emission computed tomography (SPECT). SPECT images were analysed using Neurological Statistical Image Analysis software, which has three‐dimensional stereotactic surface projections developed by Minoshima *et al*. for evaluating the spatial distribution of abnormal perfusion, to exclude other potential causes of dementia, including dementia with Lewy bodies and frontotemporal lobar degeneration, etc.^7^ A reduction in regional cerebral blood flow (rCBF) of the parietotemporal association cortex on SPECT has been recognized as a diagnostic pattern of prodromal AD.^8^

The subjects were diagnosed as having MCI due to AD according to the National Institute on Aging–Alzheimer's Association criteria,^9^ and their MMSE scores were 24 or above. Subjects were excluded from the study if they did not show any reduction in rCBF in the parietotemporal association areas. Subjects were also excluded if they had territorial or cortical infarctions, or if they showed severe white matter abnormalities in which both PVH and DWMH were grade 3 on the Fazekas scale.^10^ Cognitive functions and depressive symptoms were assessed by various neuropsychological tests, such as MMSE, Frontal Assessment Battery (FAB), Trail Making Test (TMT)‐A/B, Wechsler Memory Scale‐Revised‐Logical Memory I, category verbal fluency, and Geriatric Depression Scale (GDS)‐15.

Levels of serum CysC, 25‐hydroxyvitamin D, and homocysteine were measured using colloidal gold agglutination, radioimmunoassay, and high‐performance liquid chromatography, respectively. The levels of other cerebrovascular risk factors, including total cholesterol, low‐density lipoprotein cholesterol, glucose, hemoglobin A1c, vitamin B12, blood urea nitrogen, creatinine, estimated glomerular filtration rate, aspartate transaminase, and alanine aminotransferase were also measured.

### Magnetic resonance imaging and volumetric analysis

Brain MRI scans (3D‐T1 and T2 fluid‐attenuated inversion recovery [FLAIR] imaging) were performed using a 1.5‐tesla scanner (Magnetom; Siemens Medical Systems, Erlangen, Germany). FLAIR sequences were obtained using the following parameters: TR, 9000 ms; TE, 104 ms; TI, 2500 ms; slice thickness, 4.0 mm; and gap, 0.0 mm. For quantitative analysis of WMH volumes, FLAIR images were registered. WMH was defined as the presence of hyperintensity in the white matter area. PVH and DWMH lesions were outlined by a neurologist, using the semiautomated freeware 3D‐slicer, which is a freely available, open‐source software package for the visualization, registration, segmentation, and quantification of medical data (http://www.slicer.org).^11^ Furthermore, intracranial volumes (ICVs) were calculated on 3D‐T1 using the Voxel‐based morphometry, which was implemented in Statistical Parametric Mapping (SPM8, Wellcome Institute of Neurology, University College London, UK), and the ratios (%) of PVH and DWMH volumes to ICV were used for rating white matter abnormalities. For assessing regional PVH and DWMH (rPVH and rDWMH), rPVH and rDWMH volumes in each periventricular parcellated region within bilateral areas of the cornu frontale, pars parietalis, cornu occipitale, and pars temporalis/lobar; and within bilateral areas of the frontal, parietal, occipital and temporal lobes were calculated. These regions were manually parcellated in a template image, which was used in automated WMH extraction analysis.^12^ The parcellated regions were mapped onto individual brains using the nonlinear warping parameter calculated from the automated process. Correlations between rPVH and rDWMH volumes to ICV ratio (%) and cognitive function, and blood levels of various molecules, r were investigated. Furthermore, rPVH and rDWMH volumes were compared between MCI patients with vascular risk factors, such as HT, DM, and DL, and MCI patients without these risk factors.

### Statistical analysis

Demographic and laboratory data were calculated as means ± SD. Statistical analyses (the Student *t*‐test, Pearson test, linear multiple regression analysis, and analysis of covariance [ANCOVA]) were performed using SPSS 26.0 software.

## Results

A summary of the comparisons of subject characteristics is shown in Table 1. There were significant differences between the MCI group and the NC group with regard to scores on neuropsychological tests, such as MMSE, FAB, TMT‐B, and GDS‐15. Table 2 shows a comparison of total/regional PVH and DWMH volumes with ICV ratio between the MCI group and the NC group. In Table 2, the ratio of PVH and DWMH volumes to ICV in every region is greater in the MCI group than in the NC group, but there are no statistically significant differences. Table 3 shows that the ratio of the rPVH volume (bilateral areas of the cornu frontale, pars parietalis, and cornu occipitale) to ICV is significantly correlated with only TMT‐A/B scores and CysC level by Pearson analysis. Although the results are not shown, none of the rDWMH volumes are correlated with any of the items. Table 4 shows the correlations between rPVH and rDWMH volume ratios and the blood levels of various molecules, calculated by multiple linear regression analysis. rPVH volume (bilateral cornu frontale and left cornu occipital) and rDWMH volume (left parietal lobe) were found to correlate positively with CysC level. Table 5 shows a comparison of the ratios of total and regional PVH and DWMH volumes to ICV between MCI patients with each vascular risk factor and those without, after adjusting for age and the presence of other vascular risk factors, i.e., HT, DM, and DL, using ANCOVA. rPVH volumes (right cornu frontale, bilateral pars parietalis and cornu occipitale, and right pars temporalis) and rDWMH volumes (left frontal and parietal lobes) were significantly larger in MCI patients with DM than in those without DM, but the other risk factors showed no effect.

**Table 1:** Demographic, clinical, and blood biochemistry data of MCI and NC subjects

	MCI (*n* = 38)	NC (*n* = 10)
Sex (M/F)	13/25	5/5
Age	77.4 ± 5.6	76.5 ± 6.2
Education (years)	13.4 ± 2.3	14.4 ± 1.6
MMSE	27.3 ± 1.6*	28.7 ± 0.9
FAB	13.0 ± 2.2*	14.9 ± 2.0
TMT‐A (seconds)	52.7 ± 20.8	42.2 ± 12.6
TMT‐B (seconds)	156.9 ± 80.8*	102.5 ± 39.6
WMS‐R‐Logical Memory (immediate)	13.6 ± 6.7	15.5 ± 5.9
VF (category)	14.5 ± 3.4	15.9 ± 3.4
GDS‐15	3.8 ± 3.1*	1.7 ± 1.4
HbA1c (%)	5.9 ± 0.4	6.1 ± 0.5
T‐Cho (mg/dL)	205.0 ± 33.6	187.2 ± 17.6
LDL‐Cho (mg/dL)	113.1 ± 26.0	103.4 ± 17.6
eGFR (mL/min/1.73 m^2^)	66.2 ± 13.4	67.5 ± 18.9
Cystatin C (mg/L)	1.0 ± 0.2	1.1 ± 0.3
25(OH)VitD (ng/mL)	25.0 ± 11.9	19.9 ± 6.1
Homocysteine (nmoL/mL)	10.5 ± 3.5	9.4 ± 2.8
Systolic blood pressure (mmHg)	133.7 ± 17.4	132.2 ± 13.9
Diastolic blood pressure (mmHg)	73.7 ± 13.6	74.8 ± 11.1
Hypertension, *n* (%)	21 (55)	2 (20)
Diabetes mellitus, *n* (%)	7 (18)	3 (30)
Dyslipidemia, *n* (%)	20 (53)	4 (40)
Coronary artery disease, *n* (%)	3 (8)	1 (10)

* *P* < 0.05 between MCI and NC.

25(OH)VitD, 25‐hydroxyvitamin D; eGFR, estimated glomerular filtration rate; FAB, Frontal Assessment Battery; GDS, Geriatric Depression Scale; HbA1c, hemoglobin A1c; LDL‐Cho, low‐density lipoprotein cholesterol; MCI, mild cognitive impairment; MMSE, Mini Mental State Examination; NC, normal control; T‐Cho, total cholesterol; TMT, Trail Making Test; VF, verbal fluency; WMS‐R, Wechsler Memory Scale‐Revised.

**Table 2:** Comparison of ratios of total WMH and regional WMH volume to ICV between MCI and NC patients

Location of WMH	MCI (*n* = 38)	NC (*n* = 10)
	WMH volume to ICV ratio (%)	WMH volume to ICV ratio (%)
Total PVH	0.70 ± 0.60	0.45 ± 0.46
Total DWMH	0.30 ± 0.70	0.20 ± 0.37
L. periventricular cornu frontale	1.24 *10^−1^ ± 1.11 *10^−1^	0.85 *10^−1^ ± 0.94 *10^−1^
R. periventricular cornu frontale	1.31 *10^−1^ ± 1.14 *10^−1^	0.77 *10^−1^ ± 0.85 *10^−1^
L. periventricular pars parietalis	2.14 *10^−1^ ± 2.15 *10^−1^	1.34 *10^−1^ ± 1.83 *10^−1^
R. periventricular pars parietalis	2.16 *10^−1^ ± 2.43 *10^−1^	1.35 *10^−1^ ± 1.70 *10^−1^
L. periventricular cornu occipitale	1.29 *10^−1^ ± 1.66 *10^−1^	0.79 *10^−1^ ± 1.44 *10^−1^
R. periventricular cornu occipitale	1.34 *10^−1^ ± 1.74 *10^−1^	0.91 *10^−1^ ± 1.48 *10^−1^
L. periventricular pars temporalis	6.66 *10^−3^ ± 0.11 *10^−1^	2.84 *10^−3^ ± 5.15 *10^−3^
R. periventricular pars temporalis	5.99 *10^−3^ ± 0.11 *10^−1^	4.66 *10^−3^ ± 8.56 *10^−3^
L. frontal lobe	2.67 *10^−2^ ± 5.02 *10^−2^	1.58 *10^−2^ ± 3.09 *10^−2^
R. frontal lobe	2.73 *10^−2^ ± 4.87 *10^−2^	2.23 *10^−2^ ± 3.30 *10^−2^
L. temporal lobe	6.12 *10^−4^ ± 3.40 *10^−3^	7.34 *10^−5^ ± 2.32 *10^−4^
R. temporal lobe	9.49 *10^−4^ ± 3.71 *10^−3^	9.54 *10^−5^ ± 3.02 *10^−4^
L. parietal lobe	7.10 *10^−3^ ± 1.98 *10^−2^	1.38 *10^−3^ ± 2.17 *10^−3^
R. parietal lobe	7.69 *10^−3^ ± 2.30 *10^−2^	1.11 *10^−3^ ± 1.49 *10^−3^
L. occipital lobe	4.23 *10^−5^ ± 1.82 *10^−4^	<1.0 *10^−9^
R. occipital lobe	1.78 *10^−4^ ± 7.77 *10^−4^	<1.0 *10^−9^

DWMH, deep white matter hyperintensities; ICV, intracranial volume; L, left; MCI, mild cognitive impairment; NC, normal control; PVH, periventricular hyperintensities; R, right; WMH, white matter hyperintensities.

**Table 3:** Measurements associated with ratios of regional WMH volume to ICV in MCI patients

Measurements	L. cornu frontale vol.	R. cornu frontale vol.	L. pars parietalis vol.	R. pars parietalis vol.	L. cornu occipitale vol.	R. cornu occipitale vol.
	Coefficient/*P*	Coefficient/*P*	Coefficient/*P*	Coefficient/*P*	Coefficient/*P*	Coefficient/*P*
TMT‐A	0.37/0.03*	0.43/0.01*	0.42/0.01*	0.42/0.01*	0.38/0.03*	0.41/0.02*
TMT‐B	0.16/0.37	0.19/0.28	0.35/0.04*	0.32/0.04*	0.35/0.04*	0.35/0.04*
Cystatin C	0.40/0.01*	0.36/0.03*	0.33/0.04*	0.24/0.16	0.32/0.05*	0.29/0.07

* *P* < 0.05, significant correlation with ratio of regional WMH volume to ICV.

ICV, intracranial volume; L, left; MCI, mild cognitive impairment; R, right; TMT, Trail Making Test; WMH, white matter hyperintensities.

**Table 4:** Linear regression analyses of blood levels of molecules associated with the ratio of rPVH and rDWMH volume to ICV in MCI patients

Dependent variable	HbA1c	T‐Cho	LDL‐Cho	eGFR	Cystatin C	25(OH)VitD	Homocysteine
	Coefficient/*P*	Coefficient*/P*	Coefficient/*P*	Coefficient/*P*	Coefficient/*P*	Coefficient/*P*	Coefficient/*P*
PVH vol. ratio (L. periventricular cornu frontale)	0.130/0.392	−0.665/0.029*	0.322/0.262	−0.064/0.737	0.516/0.039*	0.184/0.240	−0.084/0.691
PVH vol. ratio (R. periventricular cornu frontale)	0.241/0.088	−0.619/0.026*	0.158/0.545	−0.179/0.311	0.456/0.046*	0.141/0.321	−0.081/0.674
PVH vol. ratio (L. periventricular pars parietalis)	0.053/0.725	−0.480/0.106	−0.006/0.984	−0.345/0.078	0.446/0.071	−0.116/0.452	−0.182/0.389
PVH vol. ratio(R. periventricular pars parietalis)	0.111/0.471	−0.605/0.048*	0.163/0.572	−0.449/0.027*	0.295/0.233	−0.044/0.780	−0.167/0.436
PVH vol. ratio (L. periventricular cornu occipitale)	0.010/0.949	−0.393/0.188	0.032/0.912	−0.307/0.120	0.584/0.022*	−0.134/0.395	−0.275/0.202
PVH vol. ratio (R. periventricular cornu occipitale)	0.057/0.690	−0.410/0.146	−0.077/0.774	−0.530/0.007**	0.349/0.135	−0.171/0.249	−0.207/0.305
PVH vol. ratio (L. periventricular pars temporalis)	−0.120/0.463	−0.572/0.076	0.193/0.529	−0.160/0.441	0.405/0.127	−0.190/0.259	−0.003/0.991
PVH vol. ratio (R. periventricular pars temporalis)	0.265/0.088	−0.863/0.006*	0.731/0.015*	−0.264/0.178	−0.154/0.526	0.121/0.440	0.477/0.031*
DWMH vol. ratio (L. frontal lobe)	0.222/0.153	−0.308/0.301	−0.039/0.891	−0.285/0.150	0.353/0.156	0.316/0.051	−0.384/0.080
DWMH vol. ratio (R. frontal lobe)	0.311/0.066	−0.499/0.124	0.145/0.639	−0.393/0.068	−0.036/0.891	0.213/0.214	0.009/0.969
DWMH vol. ratio (L. temporal lobe)	−0.095/0.599	−0.600/0.094	0.575/0.099	0.073/0.752	−0.320/0.273	0.166/0.373	0.499/0.056
DWMH vol. ratio (R. temporal lobe)	−0.123/0.487	−0.584/0.095	0.557/0.103	0.094/0.675	−0.293/0.304	0.283/0.127	0.485/0.057
DWMH vol. ratio (L. parietal lobe)	0.071/0.645	−0.247/0.407	−0.049/0.866	−0.119/0.544	0.693/0.008**	0.218/0.174	−0.496/0.027*
DWMH vol. ratio (R. parietal lobe)	0.203/0.247	−0.010/0.975	−0.366/0.268	−0.414/0.068	0.101/0.716	−0.020/0.912	−0.281/0.252
DWMH vol. ratio (L. occipital lobe)	0.050/0.797	−0.110/0.769	0.137/0.708	−0.319/0.202	−0.212/0.496	−0.111/0.579	−0.045/0.868
DWMH vol. ratio (R. occipital lobe)	0.107/0.588	0.320/0.401	−0.329/0.377	−0.196/0.434	−0.211/0.504	−0.165/0.415	0.129/0.639

* *P* < 0.05, significant correlation with blood level of the molecule.

** *P* < 0.01, significant correlation with blood level of the molecule.

25(OH)VitD, 25‐hydroxyvitamin D; DWMH, deep white matter hyperintensities; eGFR, estimated glomerular filtration rate; HbA1c, hemoglobin A1c; ICV, intracranial volume; L, left; LDL‐Cho, low‐density lipoprotein cholesterol; MCI, mild cognitive impairment; PVH, periventricular hyperintensities; R, right; T‐Cho, total cholesterol.

**Table 5:** Comparison of ratios of regional PVH and DWMH volumes to ICV between MCI patients with each vascular risk factor and those without

Ratio of total or regional WMH vol. to ICV (%)	HT (+) (*n* = 21)	HT (−) (*n* = 17)	DM (+) (*n* = 7)	DM (−) (*n* = 31)	DL (+) (*n* = 20)	DL (−) (*n* = 18)
Total PVH vol. ratio	0.80 ± 0.12	0.57 ± 0.14	1.05 ± 0.22	0.62 ± 0.10	0.61 ± 0.13	0.80 ± 0.13
Total DWMH vol. ratio	0.34 ± 0.15	0.35 ± 0.17	0.94 ± 0.26 ^#^	0.21 ± 0.12	0.28 ± 0.15	0.42 ± 0.16
PVH vol. ratio (L. periventricular cornu frontale)	1.41 *10^−1^ ± 0.25 *10^−1^	1.03 *10^−1^ ± 0.28 *10^−1^	1.73 *10^−1^ ± 0.43 *10^−1^	1.13 *10^−1^ ± 0.20 *10^−1^	1.26 *10^−1^ ± 0.25 *10^−1^	1.22 *10^−1^ ± 0.27 *10^−1^
PVH vol. ratio (R. periventricular cornu frontale)	1.46 *10^−1^ ± 0.25 *10^−1^	1.13 *10^−1^ ± 0.28 *10^−1^	2.19 *10^−1^ ± 0.43 *10^−1^ ^#^	1.11 *10^−1^ ± 0.20 *10^−1^	1.27 *10^−1^ ± 0.25 *10^−1^	1.35 *10^−1^ ± 0.27 *10^−1^
PVH vol. ratio (L. periventricular pars parietalis)	2.28 *10^−1^ ± 0.45 *10^−1^	1.98 *10^−1^ ± 0.51 *10^−1^	3.71 *10^−1^ ± 0.79 *10^−1^ ^#^	1.79 *10^−1^ ± 0.37 *10^−1^	1.79 *10^−1^ ± 0.46 *10^−1^	2.53 *10^−1^ ± 0.49 *10^−1^
PVH vol. ratio(R. periventricular pars parietalis)	2.43 *10^−1^ ± 0.49 *10^−1^	1.82 *10^−1^ ± 0.55 *10^−1^	4.54 *10^−1^ ± 0.85 *10^−1^ ^##^	1.62 *10^−1^ ± 0.40 *10^−1^	1.72 *10^−1^ ± 0.50 *10^−1^	2.64 *10^−1^ ± 0.53 *10^−1^
PVH vol. ratio (L. periventricular cornu occipitale)	1.32 *10^−1^ ± 0.34 *10^−1^	1.25 *10^−1^ ± 0.38 *10^−1^	2.65 *10^−1^ ± 0.59 *10^−1^ ^#^	0.98 *10^−1^ ± 0.27 *10^−1^	0.92 *10^−1^ ± 0.35 *10^−1^	1.69 *10^−1^ ± 0.36 *10^−1^
PVH vol. ratio (R. periventricular cornu occipitale)	1.55 *10^−1^ ± 0.36 *10^−1^	1.09 *10^−1^ ± 0.40 *10^−1^	2.86 *10^−1^ ± 0.62 *10^−1^ ^#^	1.00 *10^−1^ ± 0.29 *10^−1^	1.03 *10^−1^ ± 0.37 *10^−1^	1.69 *10^−1^ ± 0.39 *10^−1^
PVH vol. ratio (L. periventricular pars temporalis)	7.0 *10^−3^ ± 2.0 *10^−3^	6.0 *10^−3^ ± 3.0 *10^−3^	1.4 *10^−2^ ± 0.4 *10^−2^	0.5 *10^−2^ ± 0.2 *10^−2^	6.0 *10^−3^ ± 2.0 *10^−3^	8.0 *10^−3^ ± 3.0 *10^−3^
PVH vol. ratio (R. periventricular pars temporalis)	7.0 *10^−3^ ± 2.0 *10^−3^	4.0 *10^−3^ ± 2.0 *10^−3^	1.6 *10^−2^ ± 0.4 *10^−2^ ^##^	0.4 *10^−2^ ± 0.2 *10^−2^	5.0 *10^−3^ ± 2.0 *10^−3^	7.0 *10^−3^ ± 2.0 *10^−2^
DWMH vol. ratio (L. frontal lobe)	2.8 *10^−2^ ± 1.1 *10^−2^	2.5 *10^−2^ ± 1.2 *10^−2^	6.2 *10^−2^ ± 1.9 *10^−2^ ^#^	1.9 *10^−2^ ± 0.9 *10^−2^	2.9 *10^−2^ ± 1.1 *10^−2^	2.4 *10^−2^ ± 1.2 *10^−2^
DWMH vol. ratio (R. frontal lobe)	3.2 *10^−2^ ± 1.1 *10^−2^	2.2 *10^−2^ ± 1.2 *10^−2^	5.1 *10^−2^ ± 1.9 *10^−2^	2.2 *10^−2^ ± 0.9 *10^−2^	2.8 *10^−2^ ± 1.1 *10^−2^	2.7 *10^−2^ ± 1.2 *10^−2^
DWMH vol. ratio (L. temporal lobe)	1.0 *10^−3^ ± 1.0 *10^−3^	8.59 *10^−5^ ± 1.0 *10^−3^	1.0 *10^−3^ ± 1.0 *10^−3^	1.0 *10^−3^ ± 1.0 *10^−3^	<1.0*10^−6^ ± 0.001	0.001 ± 0.001
DWMH vol. ratio (R. temporal lobe)	2.0 *10^−3^ ± 1.0 *10^−3^	0.00 ± 1.0 *10^−3^	1.0 *10^−3^ ± 1.0 *10^−3^	1.0 *10^−3^ ± 1.0 *10^−3^	<1.0*10^−6^ ± 0.001	0.002 ± 0.001
DWMH vol. ratio (L. parietal lobe)	6.0 *10^−3^ ± 4.0 *10^−3^	9.0 *10^−3^ ± 5.0 *10^−3^	2.2 *10^−2^ ± 0.8 *10^−2^ ^#^	0.4 *10^−2^ ± 0.4 *10^−2^	6.0 *10^−3^ ± 4.0 *10^−3^	9.0 *10^−3^ ± 5.0 *10^−3^
DWMH vol. ratio (R. parietal lobe)	8.0 *10^−3^ ± 5.0 *10^−3^	8.0 *10^−3^ ± 6.0 *10^−3^	2.1 *10^−2^ ± 0.9 *10^−2^	0.5 *10^−2^ ± 0.4 *10^−2^	6.0 *10^−3^ ± 5.0 *10^−3^	1.0 *10^−2^ ± 6.0 *10^−3^
DWMH vol. ratio (L. occipital lobe)	8.28 *10^−5^ ± 0.00	7.59 *10^−6^ ± 0.00	7.54 *10–6 ± 0.000	5.02 *10^−5^ ± 0.000	3.51 *10^−5^ ± 0.000	5.04 *10^−5^ ± 0.000
DWMH vol. ratio (R. occipital lobe)	<1.00 *10^−6^ ± 0.000	4.48 *10^−5^ ± 0.000	6.86 *10^−6^ ± 0.000	<1.00 *10^−6^ ± 0.000	<1.0*10^−6^ ± 0.000	<1.0*10^−6^ ± 0.000

^***#***^ *P* < 0.05, significant difference between WMH volume to ICV ratio in MCI patients with DM and in those without DM.

^***##***^ *P* < 0.01, significant difference between WMH volume to ICV ratio in MCI patients with DM and in those without DM.

Values are shown as the adjusted average volume ± standard error. Adjusted covariance: age and the presence of other vascular risk factors, i.e., HT, DM, and DL.

DM, diabetes mellitus; DL, dyslipidemia; DWMH, deep white matter hyperintensities; HT, hypertension; ICV, intracranial volume; L, left; MCI, mild cognitive impairment; PVH, periventricular hyperintensities; R, right.

## Discussion

In this study, we found that rPVH and rDWMH volumes in the MCI group were not statistically significantly different from those of the NC group, although every rPVH and rDWMH volume in the MCI group was greater than that of the NC group. The association between cerebrovascular disease and AD has been reported in many recent studies, including those in which autopsies were performed.^3^, ^13^ The present study is an extension of our previous study, in which we showed the association between whole PVH volume and frontal lobe dysfunction in patients with amnestic MCI, by demonstrating the association of rWMH volumes with cognitive dysfunction and vascular risk factors.^6^ We found a significant positive correlation between rPVH volumes (bilateral areas of the cornu frontale, pars parietalis and cornu occipitale) and TMT‐A/B scores and CysC level, whereas rDWMH volumes did not correlate with any of the items. The association of rPVH volumes with attention and executive dysfunction in the present study was consistent with the results of our previous study on whole PVH and DWMH volumes.^6^ Therefore, we believe that the present results demonstrate more clearly that attention and executive dysfunction are associated more closely with PVH volume than with DWMH volume in patients with MCI. However, we have not investigated other parameters, such as visuospatial cognition. Therefore, not only attention and executive dysfunctions but also some other types of cognitive dysfunction may be associated with rWMH volumes. PVH has been reported to be affected not only by ischemic changes but also by increased fluid accumulation and reduced integrity of the ventricular ependyma, which might be associated with blood–brain barrier breakdown.^6^, ^14^, ^15^ Furthermore, the periventricular region has been reported to include functionally important cholinergic neural pathways, and PVH may exacerbate pre‐existing cholinergic deficits, particularly in AD.^6^, ^16^, ^17^ However, we do not have a reasonable explanation regarding the effect of the location of rPVH on cognition, because there have been few studies to date showing the associations of rWMH with cognitive dysfunction in MCI.^18^

We found a significant positive correlation between some rPVH volumes and serum CysC level, which was also consistent with the results of our previous study.^6^ Therefore, we believe that our present study has clearly demonstrated that CysC plays direct roles in the pathological mechanisms of the brain, such as white matter abnormalities and AD pathologies, and is not just associated with renal impairment.^6^, ^19^, ^20^, ^21^ Finally, we found that among the various vascular risk factors, only DM was significantly associated with larger rPVH volumes (right cornu frontale, bilateral pars parietalis and cornu occipitale, and right pars temporalis) and rDWMH volumes (left frontal and parietal lobes) in MCI patients, and the other risk factors had no effect, when measurements were made under well‐controlled conditions. Although associations between the location of rWMH and DM have not been clarified to date, even in diffusion tensor imaging studies,^22^ several studies have shown the association between DM and WMH volume.^23^, ^24^ Furthermore, the incidence of all types of dementia has been demonstrated to be higher in individuals with DM than in those without,^25^ and the progression of WMH is associated with accelerated cognitive decline in patients with type 2 DM.^26^, ^27^ On the other hand, a reduction in the rate of conversion from MCI to dementia, and the improvement of cognition, even in dementia patients, has been reported to be possible using the appropriate therapies and care.^28^, ^29^, ^30^ Considering the above, a better understanding of the underlying mechanism of amnestic MCI using assessments of WMH volume, serum CysC level, and vascular risk factors, particularly DM, will be important to establish appropriate treatment strategies for the prevention of the conversion from MCI to dementia.

This study has some limitations. First, the analyses were based on data obtained at one time point, namely at the initial visit. Therefore, we are planning to perform longitudinal analyses regarding the association between rWMH progression and cognitive decline. Second, we excluded subjects showing severe white matter abnormalities, and hence the results should be interpreted with caution. Third, the underlying pathology of the MCI patients was not confirmed in the present study. However, neuroimaging data were used as part of the diagnostic process. In particular, decreases in rCBF in the parietotemporal association cortex on SPECT have been recognized as a diagnostic pattern of MCI caused by AD.^8^ Therefore, we are confident that most of the MCI patients in the present series did indeed have AD pathology.

In conclusion, PVH volumes (bilateral areas of the cornu frontale, pars parietalis, and cornu occipitale) were closely associated with attention and executive dysfunction. Both serum CysC level and the presence of DM were associated with WMH volume, which suggests that CysC level and the presence of DM might be useful markers for determining treatment strategies for white matter abnormalities in amnestic MCI patients.

## Disclosure statement

The authors have no conflicts of interest to declare regarding this study.
